# *N*-(4-Meth­oxy­benzo­yl)-2-methyl­benzene­sulfonamide

**DOI:** 10.1107/S1600536814001354

**Published:** 2014-01-22

**Authors:** S. Sreenivasa, B. S. Palakshamurthy, S. Madankumar, N. K. Lokanath, P. A. Suchetan

**Affiliations:** a Department of Studies and Research in Chemistry, Tumkur University, Tumkur, Karnataka 572 103, India; b Department of Studies and Research in Physics, U.C.S., Tumkur University, Tumkur, Karnataka 572 103, India; c Department of Studies in Physics, University of Mysore, Manasagangotri, Mysore, India; d Department of Studies and Research in Chemistry, U.C.S., Tumkur University, Tumkur, Karnataka 572 103, India

## Abstract

In the title compound, C_15_H_15_NO_4_S, the dihedral angle between the aromatic rings is 80.81 (1)° and the dihedral angle between the planes defined by the S—N—C=O fragment and the sulfonyl benzene ring is 86.34 (1)°. In the extended structure, dimers related by a crystallographic twofold axis are connected by pairs of both N—H⋯O hydrogen bonds and C—H⋯O inter­actions, which generate *R*_2_^2^(8) and *R*_2_^2^(14) loops, respectively. A weak aromatic π–π stacking inter­action is also observed [centroid–centroid separation = 3.7305 (3) Å].

## Related literature   

For related structures, see: Gowda *et al.* (2010 ▶); Suchetan *et al.* (2010*a* ▶,*b* ▶, 2011 ▶); Sreenivasa *et al.* (2013 ▶, 2014 ▶).
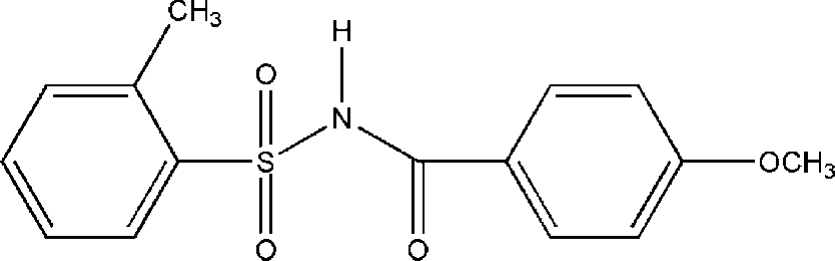


## Experimental   

### 

#### Crystal data  

C_15_H_15_NO_4_S
*M* *_r_* = 305.34
Monoclinic, 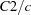

*a* = 21.807 (2) Å
*b* = 7.3521 (8) Å
*c* = 18.602 (2) Å
β = 101.211 (3)°
*V* = 2925.4 (5) Å^3^
*Z* = 8
Cu *K*α radiation
μ = 2.11 mm^−1^
*T* = 293 K
0.38 × 0.29 × 0.22 mm

#### Data collection  

Bruker APEXII CCD diffractometer
Absorption correction: multi-scan (*SADABS*; Bruker, 2009 ▶) *T*_min_ = 0.504, *T*_max_ = 0.629
16411 measured reflections
2431 independent reflections
2174 reflections with *I* > 2σ(*I*)
*R*_int_ = 0.060

#### Refinement  

*R*[*F* ^2^ > 2σ(*F* ^2^)] = 0.046
*wR*(*F* ^2^) = 0.122
*S* = 0.92
2431 reflections
196 parameters
H atoms treated by a mixture of independent and constrained refinement
Δρ_max_ = 0.34 e Å^−3^
Δρ_min_ = −0.51 e Å^−3^

### 

Data collection: *APEX2* (Bruker, 2009 ▶); cell refinement: *SAINT-Plus* (Bruker, 2009 ▶); data reduction: *SAINT-Plus*; program(s) used to solve structure: *SHELXS97* (Sheldrick, 2008 ▶); program(s) used to refine structure: *SHELXL97* (Sheldrick, 2008 ▶); molecular graphics: *Mercury* (Macrae *et al.*, 2008 ▶); software used to prepare material for publication: *SHELXL97*.

## Supplementary Material

Crystal structure: contains datablock(s) I. DOI: 10.1107/S1600536814001354/hb7188sup1.cif

Structure factors: contains datablock(s) I. DOI: 10.1107/S1600536814001354/hb7188Isup2.hkl

Supporting information file. DOI: 10.1107/S1600536814001354/hb7188Isup3.cml

CCDC reference:

Additional supporting information:  crystallographic information; 3D view; checkCIF report

## Figures and Tables

**Table 1:** Hydrogen-bond geometry (Å, °)

*D*—H⋯*A*	*D*—H	H⋯*A*	*D*⋯*A*	*D*—H⋯*A*
N1—H1⋯O2^i^	0.81 (3)	2.16 (3)	2.917 (2)	164 (3)
C13—H13⋯O2^i^	0.93	2.56	3.288 (3)	136

Symmetry code: (i) 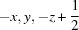.

**Fig. 1.**
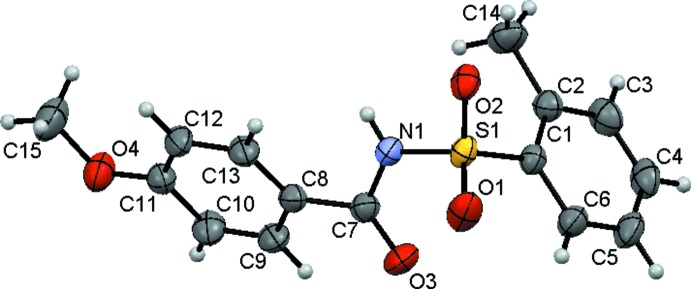
Molecular structure of the title compound, showing displacement ellipsoids drawn at the 50% probability level.

**Fig. 2.**
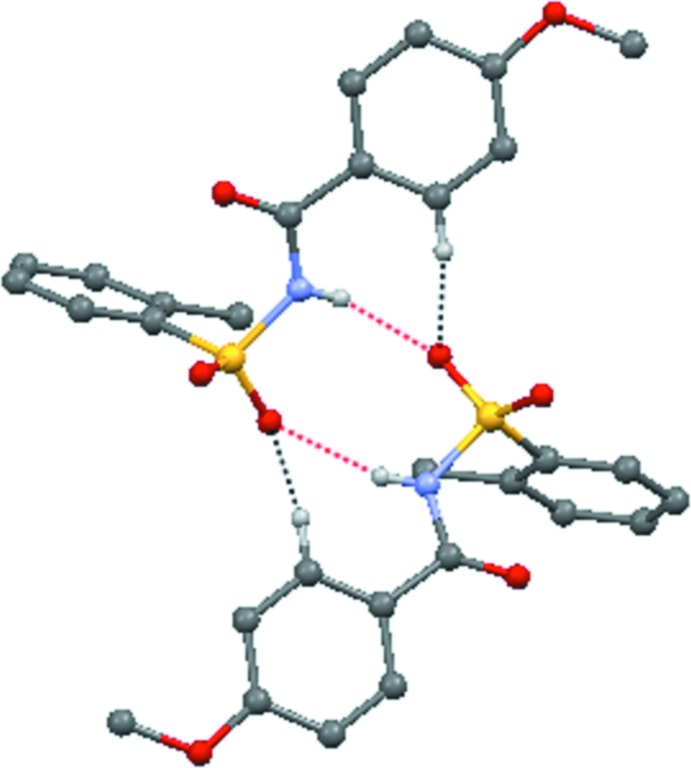
Formation of R22(8) and R22(14) rings in I.

**Fig. 3.**
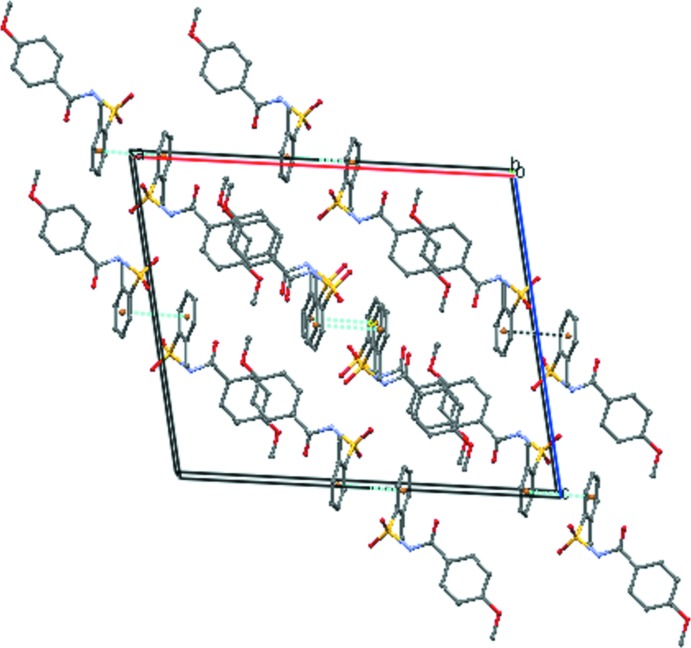
π···π interaction observed in the crystal structure. Cg is the centroid of the methylphenyl ring.

## supplementary crystallographic information

### 1. Introduction

As a part of our continued efforts to study the crystal structures of N-(aroyl)-aryl­sulfonamides (Sreenivasa *et al.*, 2014), we report here the crystal structure of the title compound (I) (Fig 1).

### 2.1. Synthesis and crystallization

The title compound (I) was prepared by refluxing a mixture of 4-meth­oxy­benzoic acid, 2-methyl­benzene­sulfonamide and phospho­rous oxychloride (POCl_3_) for 2 h on a water bath. The resultant mixture was cooled and poured into ice cold water. The solid obtained was filtered and washed thoroughly with water and then dissolved in sodium bicarbonate solution. The compound was later reprecipitated by acidifying the filtered solution with dilute HCl. The compound obtained was filtered and later dried (Melting point: 447 K).

Colorless prisms of (I) were obtained from a slow evaporation of its aqueous methano­lic solution at room temperature.

### 2.2. Refinement

The H atom of the NH group was located in a difference map and later refined freely. The other H atoms were positioned with idealized geometry using a riding model with C—H = 0.93-0.96 Å. All H atoms were refined with isotropic displacement parameters (set to 1.2-1.5 times of the U eq of the parent atom).

### 3. Results and discussion

In I, the dihedral angle between the two aromatic rings is 80.81 (1)°. Compared to this, the dihedral angle is 73.9 (1)° in N-(benzoyl)-2-methyl­benzene­sulfonamide (II, Suchetan *et al.*, 2010*a*), 89.4 (1)° and 82.4 (1)° respectively in the two molecules in the asymmetric unit of N-(4-chloro­benzoyl)-2-methyl­benzene­sulfonamide (III, Suchetan *et al*., 2010*b*), 88.1 (1)° and 83.5 (1)° respectively in the two molecules in the asymmetric unit of N-(4-methyl­benzoyl)-2-methyl­benzene­sulfonamide (IV, Gowda *et al.*, 2010) and 83.8 (2)° in N-(4-nitro­benzoyl)-2-methyl­benzene­sulfonamide (V, Suchetan *et al.*, 2011). This shows that introducing a substituent into the para position of the benzoyl ring of II correlates with a increase of the dihedral angle between the aromatic rings. In contrast to this, the dihedral angle is small in N-(4-meth­oxy-benzoyl)-benzene­sulfonamide (VI, Sreenivasa *et al.*, 2014) and N-(4-meth­oxy­benzoyl)-4-methyl­benzene­sulfonamide (VII, Sreenivasa *et al.*, 2013), the dihedral angle being respectively 69.81 (1)° and 78.62 (16)° in VI and VII. Further, the molecule is twisted at the S atom, the dihedral angle between the planes defined by the S—N—C=O segment in the central chain and the sulfonyl benzene ring being 86.34 (1)°.

The supra­molecular architecture of I is built in three stages. In the first stage, the molecules are linked into dimers by a crystallographic twofold axis through strong N1—H1···O2 hydrogen bonds, thus generating R_2_^2^(8) rings (Figure 2). These dimers in the second stage are linked through an additional C13—H13···O2 inter­action (Figure 2) forming R_2_^2^(14) ring motif. In the third stage, π(methyl­phenyl) ···π(methyl­phenyl) inter­actions stabilize the structure, Cg(methyl­phenyl) ···Cg(methyl­phenyl) distance being 3.7305 (3)Å (Figure 3). The geometries and symmetry operations of various inter­actions are shown in Table 1.

### Crystal data

**Table:**

C_15_H_15_NO_4_S	Prism
*M**_r_* = 305.34	*D*_x_ = 1.387 Mg m^−^^3^
Monoclinic, *C*2/*c*	Melting point: 447 K
Hall symbol: -C 2yc	Cu *K*α radiation, λ = 1.54178 Å
*a* = 21.807 (2) Å	Cell parameters from 25 reflections
*b* = 7.3521 (8) Å	θ = 4.1–64.7°
*c* = 18.602 (2) Å	µ = 2.11 mm^−^^1^
β = 101.211 (3)°	*T* = 293 K
*V* = 2925.4 (5) Å^3^	Prism, colourless
*Z* = 8	0.38 × 0.29 × 0.22 mm
*F*(000) = 1280	

### Data collection

**Table:**

Bruker APEXII CCD diffractometer	2431 independent reflections
Radiation source: fine-focus sealed tube	2174 reflections with *I* > 2σ(*I*)
Graphite monochromator	*R*_int_ = 0.060
phi and φ scans	θ_max_ = 64.7°, θ_min_ = 4.1°
Absorption correction: multi-scan (*SADABS*; Bruker, 2009)	*h* = −25→24
*T*_min_ = 0.504, *T*_max_ = 0.629	*k* = −7→8
16411 measured reflections	*l* = −20→21

### Refinement

**Table:**

Refinement on *F*^2^	Primary atom site location: structure-invariant direct methods
Least-squares matrix: full	Secondary atom site location: difference Fourier map
*R*[*F*^2^ > 2σ(*F*^2^)] = 0.046	Hydrogen site location: inferred from neighbouring sites
*wR*(*F*^2^) = 0.122	H atoms treated by a mixture of independent and constrained refinement
*S* = 0.92	*w* = 1/[σ^2^(*F*_o_^2^) + (0.0923*P*)^2^ + 2.3453*P*] where *P* = (*F*_o_^2^ + 2*F*_c_^2^)/3
2431 reflections	(Δ/σ)_max_ < 0.001
196 parameters	Δρ_max_ = 0.34 e Å^−^^3^
0 restraints	Δρ_min_ = −0.51 e Å^−^^3^

### Special details

**Table:**

Geometry. All s.u.'s (except the s.u. in the dihedral angle between two l.s. planes) are estimated using the full covariance matrix. The cell s.u.'s are taken into account individually in the estimation of s.u.'s in distances, angles and torsion angles; correlations between s.u.'s in cell parameters are only used when they are defined by crystal symmetry. An approximate (isotropic) treatment of cell s.u.'s is used for estimating s.u.'s involving l.s. planes.
Refinement. Refinement of *F*^2^ against ALL reflections. The weighted *R*-factor *wR* and goodness of fit *S* are based on *F*^2^, conventional *R*-factors *R* are based on *F*, with *F* set to zero for negative *F*^2^. The threshold expression of *F*^2^ > 2σ(*F*^2^) is used only for calculating *R*-factors(gt) *etc*. and is not relevant to the choice of reflections for refinement. *R*-factors based on *F*^2^ are statistically about twice as large as those based on *F*, and *R*- factors based on ALL data will be even larger.

### Fractional atomic coordinates and isotropic or equivalent isotropic displacement parameters (Å^2^)

**Table:**

	*x*	*y*	*z*	*U*_iso_*/*U*_eq_	
H1	0.0708 (12)	0.197 (3)	0.2813 (15)	0.051 (7)*	
S1	0.02647 (2)	0.20056 (7)	0.37394 (2)	0.0326 (2)	
O2	−0.02356 (6)	0.2872 (2)	0.32370 (7)	0.0408 (4)	
O1	0.01308 (8)	0.0360 (2)	0.40868 (8)	0.0499 (4)	
O3	0.15501 (7)	0.0483 (2)	0.41305 (7)	0.0464 (4)	
C8	0.17364 (9)	0.0372 (3)	0.29109 (9)	0.0335 (4)	
N1	0.07872 (7)	0.1589 (2)	0.32300 (8)	0.0346 (4)	
O4	0.28653 (7)	−0.0475 (2)	0.13921 (8)	0.0512 (4)	
C13	0.14633 (9)	0.0201 (3)	0.21740 (10)	0.0395 (5)	
H13	0.1031	0.0286	0.2031	0.047*	
C6	0.07042 (10)	0.2955 (3)	0.51353 (10)	0.0406 (5)	
H6	0.0606	0.1764	0.5238	0.049*	
C12	0.18241 (10)	−0.0093 (3)	0.16511 (10)	0.0399 (5)	
H12	0.1636	−0.0204	0.1160	0.048*	
C2	0.07397 (9)	0.5358 (3)	0.42381 (10)	0.0372 (5)	
C1	0.06024 (8)	0.3584 (3)	0.44137 (9)	0.0317 (4)	
C7	0.13675 (9)	0.0789 (3)	0.34846 (9)	0.0341 (4)	
C11	0.24672 (10)	−0.0224 (3)	0.18620 (10)	0.0372 (5)	
C10	0.27437 (10)	−0.0116 (3)	0.26017 (10)	0.0439 (5)	
H10	0.3175	−0.0233	0.2746	0.053*	
C3	0.09891 (10)	0.6503 (3)	0.48231 (12)	0.0476 (5)	
H3	0.1087	0.7699	0.4729	0.057*	
C4	0.10915 (10)	0.5888 (3)	0.55374 (11)	0.0488 (6)	
H4	0.1257	0.6677	0.5917	0.059*	
C9	0.23797 (10)	0.0164 (3)	0.31184 (10)	0.0402 (5)	
H9	0.2566	0.0214	0.3612	0.048*	
C5	0.09543 (11)	0.4140 (4)	0.56954 (10)	0.0484 (6)	
H5	0.1029	0.3744	0.6179	0.058*	
C14	0.06366 (13)	0.6127 (3)	0.34685 (11)	0.0543 (6)	
H14A	0.0891	0.5479	0.3188	0.082*	
H14B	0.0749	0.7392	0.3489	0.082*	
H14C	0.0204	0.5999	0.3240	0.082*	
C15	0.25999 (13)	−0.0489 (5)	0.06265 (12)	0.0653 (8)	
H15A	0.2278	−0.1398	0.0529	0.098*	
H15B	0.2920	−0.0762	0.0354	0.098*	
H15C	0.2423	0.0682	0.0483	0.098*	

### Atomic displacement parameters (Å^2^)

**Table:**

	*U*^11^	*U*^22^	*U*^33^	*U*^12^	*U*^13^	*U*^23^
S1	0.0365 (3)	0.0348 (3)	0.0268 (3)	−0.00716 (18)	0.00702 (19)	−0.00524 (15)
O2	0.0324 (7)	0.0554 (10)	0.0325 (6)	0.0007 (6)	0.0010 (5)	−0.0104 (6)
O1	0.0681 (10)	0.0406 (10)	0.0444 (8)	−0.0208 (8)	0.0191 (7)	−0.0039 (6)
O3	0.0543 (9)	0.0550 (10)	0.0273 (7)	0.0034 (7)	0.0017 (6)	0.0049 (6)
C8	0.0386 (10)	0.0296 (11)	0.0303 (9)	0.0028 (8)	0.0019 (7)	−0.0019 (7)
N1	0.0392 (9)	0.0399 (10)	0.0253 (7)	0.0049 (7)	0.0074 (6)	0.0007 (6)
O4	0.0439 (8)	0.0725 (12)	0.0379 (7)	0.0054 (8)	0.0095 (6)	−0.0081 (7)
C13	0.0351 (10)	0.0450 (13)	0.0345 (10)	0.0073 (9)	−0.0032 (8)	−0.0057 (8)
C6	0.0489 (11)	0.0422 (13)	0.0307 (9)	−0.0017 (9)	0.0075 (8)	−0.0004 (8)
C12	0.0430 (11)	0.0445 (12)	0.0287 (9)	0.0080 (9)	−0.0013 (7)	−0.0074 (8)
C2	0.0403 (10)	0.0341 (12)	0.0357 (9)	0.0005 (9)	0.0034 (7)	−0.0032 (8)
C1	0.0326 (9)	0.0352 (11)	0.0266 (8)	−0.0011 (8)	0.0046 (6)	−0.0040 (7)
C7	0.0403 (10)	0.0304 (11)	0.0302 (9)	−0.0022 (8)	0.0035 (7)	−0.0012 (7)
C11	0.0396 (10)	0.0358 (12)	0.0356 (9)	0.0046 (8)	0.0059 (8)	−0.0039 (7)
C10	0.0332 (10)	0.0562 (15)	0.0388 (10)	0.0049 (9)	−0.0020 (8)	−0.0069 (9)
C3	0.0514 (12)	0.0351 (12)	0.0534 (12)	−0.0048 (10)	0.0030 (9)	−0.0116 (9)
C4	0.0499 (12)	0.0511 (15)	0.0420 (11)	−0.0006 (11)	0.0004 (9)	−0.0205 (9)
C9	0.0414 (11)	0.0449 (13)	0.0301 (9)	0.0061 (9)	−0.0039 (7)	−0.0031 (8)
C5	0.0545 (12)	0.0619 (16)	0.0267 (9)	0.0014 (11)	0.0027 (8)	−0.0083 (9)
C14	0.0776 (16)	0.0396 (14)	0.0432 (11)	−0.0074 (12)	0.0051 (10)	0.0074 (9)
C15	0.0629 (15)	0.099 (2)	0.0353 (11)	0.0046 (15)	0.0129 (10)	−0.0059 (11)

### Geometric parameters (Å, º)

**Table:**

S1—O1	1.4283 (15)	C2—C1	1.391 (3)
S1—O2	1.4398 (14)	C2—C3	1.399 (3)
S1—N1	1.6461 (16)	C2—C14	1.515 (3)
S1—C1	1.7613 (18)	C11—C10	1.393 (3)
O3—C7	1.211 (2)	C10—C9	1.376 (3)
C8—C13	1.390 (2)	C10—H10	0.9300
C8—C9	1.389 (3)	C3—C4	1.380 (3)
C8—C7	1.488 (3)	C3—H3	0.9300
N1—C7	1.392 (2)	C4—C5	1.364 (4)
N1—H1	0.81 (3)	C4—H4	0.9300
O4—C11	1.359 (2)	C9—H9	0.9300
O4—C15	1.429 (3)	C5—H5	0.9300
C13—C12	1.382 (3)	C14—H14A	0.9600
C13—H13	0.9300	C14—H14B	0.9600
C6—C5	1.386 (3)	C14—H14C	0.9600
C6—C1	1.396 (3)	C15—H15A	0.9600
C6—H6	0.9300	C15—H15B	0.9600
C12—C11	1.384 (3)	C15—H15C	0.9600
C12—H12	0.9300		
			
O1—S1—O2	118.20 (10)	O4—C11—C12	124.46 (17)
O1—S1—N1	109.14 (9)	O4—C11—C10	115.78 (18)
O2—S1—N1	103.34 (8)	C12—C11—C10	119.75 (18)
O1—S1—C1	109.25 (9)	C9—C10—C11	120.05 (19)
O2—S1—C1	109.27 (9)	C9—C10—H10	120.0
N1—S1—C1	107.00 (8)	C11—C10—H10	120.0
C13—C8—C9	118.68 (18)	C4—C3—C2	121.2 (2)
C13—C8—C7	122.57 (18)	C4—C3—H3	119.4
C9—C8—C7	118.75 (16)	C2—C3—H3	119.4
C7—N1—S1	124.60 (13)	C5—C4—C3	120.98 (19)
C7—N1—H1	118.8 (19)	C5—C4—H4	119.5
S1—N1—H1	116.3 (19)	C3—C4—H4	119.5
C11—O4—C15	117.14 (17)	C10—C9—C8	120.73 (17)
C12—C13—C8	121.00 (18)	C10—C9—H9	119.6
C12—C13—H13	119.5	C8—C9—H9	119.6
C8—C13—H13	119.5	C4—C5—C6	120.05 (19)
C5—C6—C1	118.7 (2)	C4—C5—H5	120.0
C5—C6—H6	120.6	C6—C5—H5	120.0
C1—C6—H6	120.6	C2—C14—H14A	109.5
C13—C12—C11	119.69 (17)	C2—C14—H14B	109.5
C13—C12—H12	120.2	H14A—C14—H14B	109.5
C11—C12—H12	120.2	C2—C14—H14C	109.5
C1—C2—C3	116.76 (18)	H14A—C14—H14C	109.5
C1—C2—C14	124.95 (17)	H14B—C14—H14C	109.5
C3—C2—C14	118.3 (2)	O4—C15—H15A	109.5
C2—C1—C6	122.33 (17)	O4—C15—H15B	109.5
C2—C1—S1	121.98 (13)	H15A—C15—H15B	109.5
C6—C1—S1	115.67 (16)	O4—C15—H15C	109.5
O3—C7—N1	121.18 (17)	H15A—C15—H15C	109.5
O3—C7—C8	123.64 (18)	H15B—C15—H15C	109.5
N1—C7—C8	115.18 (15)		
			
O1—S1—N1—C7	−55.03 (19)	S1—N1—C7—C8	174.04 (14)
O2—S1—N1—C7	178.35 (16)	C13—C8—C7—O3	159.9 (2)
C1—S1—N1—C7	63.07 (19)	C9—C8—C7—O3	−20.8 (3)
C9—C8—C13—C12	−2.7 (3)	C13—C8—C7—N1	−20.8 (3)
C7—C8—C13—C12	176.6 (2)	C9—C8—C7—N1	158.54 (19)
C8—C13—C12—C11	0.1 (3)	C15—O4—C11—C12	4.1 (3)
C3—C2—C1—C6	0.1 (3)	C15—O4—C11—C10	−176.6 (2)
C14—C2—C1—C6	−179.6 (2)	C13—C12—C11—O4	−178.6 (2)
C3—C2—C1—S1	178.06 (16)	C13—C12—C11—C10	2.1 (3)
C14—C2—C1—S1	−1.6 (3)	O4—C11—C10—C9	179.1 (2)
C5—C6—C1—C2	−0.4 (3)	C12—C11—C10—C9	−1.6 (4)
C5—C6—C1—S1	−178.41 (16)	C1—C2—C3—C4	0.0 (3)
O1—S1—C1—C2	−175.20 (16)	C14—C2—C3—C4	179.7 (2)
O2—S1—C1—C2	−44.48 (18)	C2—C3—C4—C5	0.2 (4)
N1—S1—C1—C2	66.76 (18)	C11—C10—C9—C8	−1.1 (4)
O1—S1—C1—C6	2.85 (18)	C13—C8—C9—C10	3.2 (3)
O2—S1—C1—C6	133.58 (15)	C7—C8—C9—C10	−176.1 (2)
N1—S1—C1—C6	−115.18 (16)	C3—C4—C5—C6	−0.4 (3)
S1—N1—C7—O3	−6.6 (3)	C1—C6—C5—C4	0.5 (3)

### Hydrogen-bond geometry (Å, º)

**Table:**

*D*—H···*A*	*D*—H	H···*A*	*D*···*A*	*D*—H···*A*
N1—H1···O2^i^	0.81 (3)	2.16 (3)	2.917 (2)	164 (3)
C13—H13···O2^i^	0.93	2.56	3.288 (3)	136

Symmetry code: (i) −*x*, *y*, −*z*+1/2.
